# Impact of NRAS Mutations on the Diagnosis of Follicular Neoplasm of the Thyroid

**DOI:** 10.1155/2014/289834

**Published:** 2014-08-31

**Authors:** Ja-Seong Bae, Seung Kyu Choi, Sora Jeon, Yourha Kim, Sohee Lee, Youn Soo Lee, Chan Kwon Jung

**Affiliations:** ^1^Department of Surgery, College of Medicine, The Catholic University of Korea, Seoul 137-701, Republic of Korea; ^2^Department of Hospital Pathology, College of Medicine, The Catholic University of Korea, 222 Banpodaero, Seocho-gu, Seoul 137-701, Republic of Korea; ^3^Department of Pathology, College of Medicine, Dankook University, Cheonan-si, Chungnam 330-714, Republic of Korea

## Abstract

*Background*. Most patients with a preoperative diagnosis of thyroid follicular neoplasm (FN) undergo diagnostic surgery to determine whether the nodule is benign or malignant. Point mutations at *NRAS* codon 61 are the most common mutations observed in FN. However, the clinical significance of *NRAS* mutation remains unclear. *Methods*. From 2012 to 2013, 123 consecutive patients undergoing thyroidectomy for FN were evaluated prospectively. Molecular analyses for *NRAS* codon 61 were performed with pyrosequencing. *Results*. The overall malignancy rate in FN was 48.8% (60/123). Of 123 FNs, 33 (26.8%) were positive for the *NRAS* mutation. The sensitivity, specificity, positive predictive value, and negative predictive value of a *NRAS* mutation-positive FN specimen to predict malignancy were 37%, 83%, 67%, and 58%, respectively. Patients with a *NRAS*-positive FN had a higher malignancy rate in additional thyroid nodules beyond the FN than patients with a NRAS-negative FN. The overall malignancy rate of patients with a NRAS-positive FN was significantly higher than that of patients with a NRAS-negative FN (79% versus 52%; *P* = 0.008). *Conclusions*. Determining NRAS mutation status in FN helps to improve the accuracy of thyroid cancer diagnosis and to predict cancer risk in accompanying thyroid nodules.

## 1. Introduction

A follicular neoplasm (FN) is a thyroid nodule that can be cancerous or noncancerous, a follicular carcinoma or a follicular adenoma, respectively. The rate of malignancy is 20–30% in nodules with FN [1, 2]. The definitive morphological diagnosis of follicular thyroid carcinoma (FTC) relies on a demonstration of capsular or vascular invasion after surgery. Many patients with follicular adenoma are operated on unnecessarily because it is not possible to discriminate between benign and malignant FN with a preoperative fine needle aspiration biopsy (FNAB) or a core needle biopsy (CNB) [3].

In recent years, molecular-based diagnostic markers, such as* BRAF*,* RAS*,* PAX8-PPAR* *γ* , or* RET-PTC* mutations, have improved the diagnosis of thyroid nodules [4]. The* BRAF* V600E mutation is the most common genetic alteration in thyroid tumorigenesis and has been observed in ~29–83% of papillary thyroid carcinoma (PTC) [5–7].* RAS* mutations are the second most common genetic alteration in thyroid tumors. Recent studies reported that 10–20% of PTC and 40–50% of FTC harbor* RAS* mutations [8, 9]. They have been associated with poor prognoses and tumor dedifferentiation [10, 11]. The* RAS* genes consist of three families:* NRAS*,* HRAS*, and* KRAS*.* RAS* point mutations mostly occur in codons 12, 13, and 61 [12, 13]. The* NRAS* mutation at codon 61 accounted for 67–88% of all* RAS* mutations [14, 15].* NRAS *mutations were more common in conventional FNs than Hürthle-cell neoplasms (HCNs) [14]. However, the diagnostic importance of* RAS* mutations has been ambiguous because these mutations are found not only in thyroid cancers but also in histologically benign nodules, including follicular adenomas and nodular hyperplasia [15].

In the present study, we analyzed the clinical significance and diagnostic utility of* NRAS* mutations in patients who underwent surgery for FN.

## 2. Subjects and Methods

### 2.1. Patient Population and Inclusion Criteria

This study was performed under Institutional Review Board approval at Seoul St. Mary's Hospital, the Catholic University of Korea. We analyzed the prospectively collected records associated with 123 patients who had a preoperative diagnosis of FN and then underwent surgery from 2012 to 2013. FNAB (*n* = 47) or CNB (*n* = 76) was performed preoperatively to diagnose the thyroid nodules. When thyroid surgery was indicated, a diagnostic lobectomy and isthmusectomy were recommended. A completion thyroidectomy was offered to patients with a surgically proven FTC with vascular invasion regardless of tumor size or Hürthle cell carcinoma after detailed counseling. All samples were rereviewed by a pathologist who specializes in thyroid pathology (CKJ). The formal diagnosis was made according to the World Health Organization classification of thyroid cancer.

### 2.2. NRAS Mutation Analysis

DNA was extracted from unstained paraffin-embedded tissue sections of surgical resection specimens using the QIAamp DNA FFPE Tissue Kit (Qiagen, Hilden, Germany) after manual microdissection. After PCR using primers targeting codon 61 using the PyroMark PCR Kit (Qiagen), pyrosequencing was performed according to standard procedures using the NRAS Pyro Kit (Qiagen) and PyroMark Q24 System (Qiagen). Program outputs were analyzed with the PyroMark Q24 software (version 2.0.6; Qiagen) using allele quantification mode. The* NRAS* mutation status was considered positive if the mutant allele percentage was 5% or more.

### 2.3. Statistical Analysis

Statistical analyses were performed with the SPSS software (version 18.0; SPSS, Chicago, IL, USA). The *χ* ^2^ test was used to compare proportions between categorical variables and the *t*-test was used to compare means between groups. Probability values < 0.05 were considered to indicate statistical significance.

## 3. Results

### 3.1. Surgical Results of FNs

**Figure 1:**
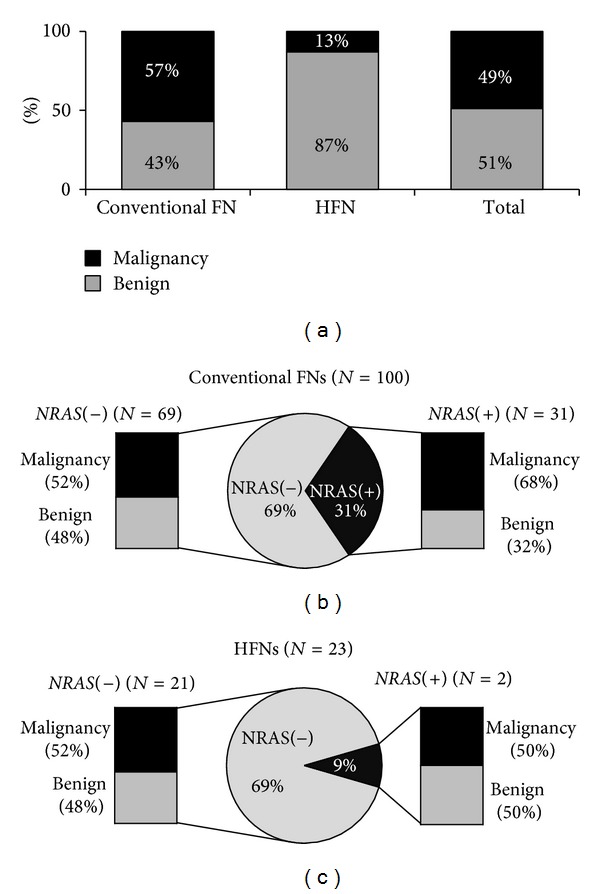
(a) Postoperative surgical pathology results of 123 thyroid nodules with a preoperative diagnosis of follicular neoplasm (FN) according to the histological subtypes. (b) Surgical results and* NRAS* mutation status of the 100 conventional FNs. (c) Surgical results and* NRAS* mutation status of the 23 Hürthle cell neoplasms (HFNs).

In total, the 123 FNs consisted of 100 conventional and 23 HCN subtypes (Figure 1). Malignancy was histologically proven in 57 of 100 conventional FNs and 3 of 23 HCNs. The overall malignancy rate was 48.8% (60/123 FNs). The malignancy rate was higher in conventional FN than in HCN (57% versus 13%; *P* < 0.001). Of 123 FNs, 33 harbored* NRAS* mutations. The mutation rate was higher in conventional FN than in HCN (31% versus 9%; *P* = 0.029) (Figure 1).

### 3.2. Clinicopathological Features and NRAS Mutation Status of FNs

**Figure 2:**
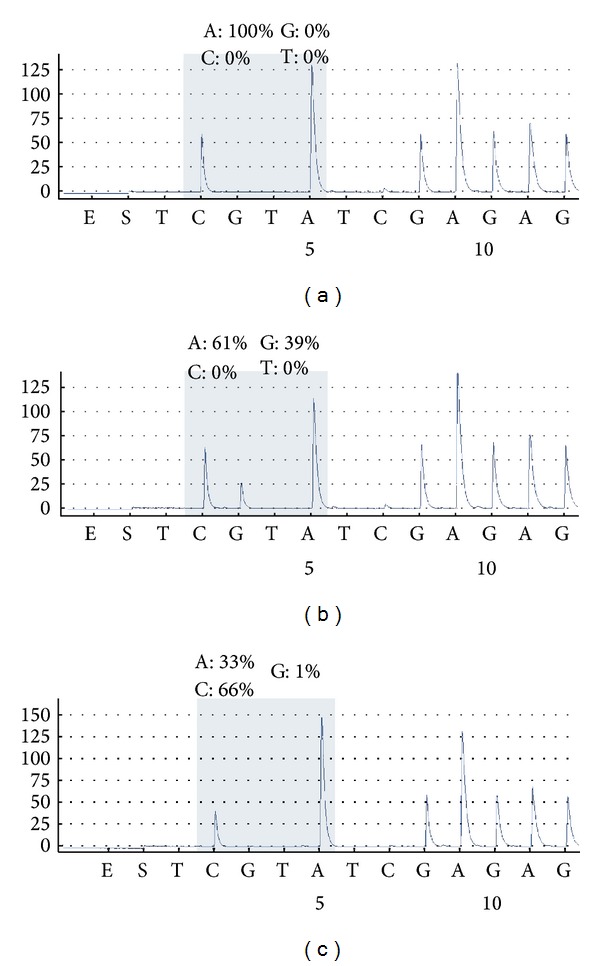
Analysis of* NRAS* mutation by pyrosequencing. Representative images of wild type (a),* NRAS* Q61R (c.182A>G) (b), and* NRAS* Q61K (c.181C>A) (c).

Of 123 FNs, 33 (26.8%) harbored* NRAS *mutations.* NRAS* Q61R (c.182A>G) mutants were found in 26 cases. Seven FNs harbored the* NRAS* Q61K (c.181C>A) mutation (Figure 2). The mutation rate was higher in conventional FN than in HCN (31% versus 9%; *P* = 0.029). The frequency of* NRAS* mutation according to the histological subtype of FN is summarized in Figure 1. The age of patients with FN ranged from 16 to 83 years (mean 48.7 ± 14.1). Age and gender were not significantly correlated with the* NRAS* mutation status (Table 1). Tumor size was larger in FNs without* NRAS* mutations (*P* < 0.001) (Table 1).

### 3.3. Risk of Malignancy in Patients with a NRAS Mutation-Positive FN

*NRAS *mutations were found more frequently in malignant than in benign nodules (36.7% versus 17.5%; *P* = 0.016). The sensitivity, specificity, positive predictive value, and negative predictive value of a* NRAS* mutation-positive FN specimen to predict malignancy were 37%, 83%, 67%, and 58%, respectively. Of all 123 patients, 60 had one or more additional thyroid nodules in addition to FN. Among the 60 patients with additional nodules, 24 had malignancy diagnosed incidentally during FN treatment. Finally, 73 of 123 patients had malignant nodules after surgery. There was a tendency that the patients with incidental malignancy in addition to FN had a higher rate of* NRAS* mutation in their FN than those with additional benign nodules (50% versus 28%; *P* = 0.080; Table 1). When we considered patients with a malignant nodule diagnosed from FN and/or another nodule as finally having thyroid malignancy, 26 of 33 (79%) patients with a* NRAS*-positive FN had thyroid malignancy whereas 47 of 90 (52%) patients with a* NRAS*-negative FN had malignancy (Table 1).

### 3.4. Subanalysis of 60 Patients Having Histologically Proven Thyroid Cancers

Of a total of 123 patients with a preoperative diagnosis of FN, 60 were proven to have malignant FN after thyroid surgery. We conducted a subanalysis of 60 thyroid cancer patients to examine the clinicopathologic significance of* NRAS* mutation in thyroid cancer (Table 2). The age of the thyroid cancer patients ranged from 24 to 81 years (38 females, 22 males). The 60 thyroid cancers consisted of 37 (62%) encapsulated follicular variant of papillary thyroid cancers (FVPTCs), 6 (10%) infiltrative FVPTCs, 3 (5%) classic PTCs, 13 (22%) FTCs, one widely invasive, 12 minimally invasive tumors, and 1 (2%) poorly differentiated carcinoma. Histological evaluations revealed the invasion of tumor capsules in 34 tumors and vascular invasion in nine tumors. No significant difference was observed in the frequency of* NRAS* mutations in terms of capsular invasion or vascular invasion. Tumor size was significantly larger in cancers without* NRAS* mutations than in those with mutations (*P* = 0.012; Table 2).

### 3.5. Subanalysis of 63 Patients with Histologically Proven Benign Thyroid Lesions

Of a total of 123 patients with a preoperative diagnosis of FN, 63 were proven to have benign thyroid lesions after thyroid surgery (Table 3). We further analyzed the relationship between* NRAS* mutation and clinicopathological features in 63 patients with benign nodules. The age of patients with benign thyroid lesions ranged from 16 to 83 (54 females, 9 males). The 63 benign thyroid lesions included 21 follicular adenomas and 12 nodular hyperplasias. Age and gender did not correlate with* NRAS* mutation status.* NRAS*-mutated benign tumors had significantly smaller tumor size than benign lesions without* NRAS* mutations (*P* = 0.010; Table 3).

### 3.6. NRAS Mutation Allele Frequency

All* NRAS* mutations found in the tumor samples were heterozygous somatic mutations. The* NRAS* mutation allele frequency ranged from 14% to 46% (median 33%), corresponding to 28–92% of cells with a heterozygous mutation. There was no difference in* NRAS* mutation allele frequencies between benign and malignant tumors (data not shown).

## 4. Discussion

We found that the rate of confirmed malignancy was 48.8% after surgery of the FNs and FVPTC was the most common type among the malignancies. The* NRAS* mutation rate was 26.8% in all FNs.* NRAS* mutations were more frequent in malignant FNs. Positive and negative predictive values of* NRAS* mutation testing in FNs for predicting malignancy were 67% and 58%, respectively. These results were consistent with those of previous studies [8, 15, 16]. Interestingly, the rate of malignancy in additional nodules coexisting with a FN was higher in patients with a* NRAS* mutation-positive FN than in those with a* NRAS* mutation-negative FN. Our results showed that overall malignancy rate in patients with a* NRAS* mutation-positive FN was 78.8%. Gupta et al. found that the presence of* RAS* mutations in thyroid FNAB specimens was related with an increased incidence of bilateral thyroid cancer and suggested that total thyroidectomy in patients with a RAS mutation-positive nodule is a more appropriate surgical option [17].

We observed that most thyroid cancers positive for* NRAS* mutation were encapsulated FVPTC and 2 (15%) of 13 FTCs carried the mutation (Table 2). These findings are consistent with a recent study which found that 13 of 14* NRAS*-positive thyroid cancers were FVPTC and one of them was FTC [18]. Pathologic diagnosis of follicular adenoma and FTC essentially requires the lack of nuclear features of PTC. PTC shows nuclear clearing, irregularity, grooves and pseudoinclusions, but the nuclei of follicular adenoma and FTC are round, regular, and not clear. Even so, the differential diagnosis of follicular adenoma and FTC from encapsulated FVPTC can be difficult even for experienced pathologists [19, 20]. The diagnostic threshold for FVPTC has been lowered over the past two decades [19, 20]. The evolution of criteria that more broadly define the pathologic diagnosis of PTC is partly responsible for a higher proportion of FVPTC rather than FTC diagnosed in our study.

Few studies have examined the role of* NRAS* mutation as a prognostic factor in FN. Jang et al. recently reported that* NRAS* mutations at codon 61 mutation were associated significantly with the presence of a distant metastasis in FTC [21]. Fukahori et al. also found that* NRAS* codon 61 mutations in 58 FTC patients were related with distant metastasis but not with recurrence or overall survival [16]. Volante et al. suggested that RAS mutations were associated with shorter overall survival rate in poorly differentiated cancers [10].

Our study showed that encapsulated FVPTCs were the most common phenotype in malignant FNs.* NRAS* mutations were found in 49% of encapsulated FVPTCs. This rate is similar to the overall RAS mutation frequencies, from 18% to 48% in follicular adenomas and from 23% to 57% in FTCs [8, 16, 22–24]. However, infiltrative FVPTCs had a lower frequency of* NRAS* mutations.

We observed that most of the tumor samples (~80%) had more than 25% of* NRAS* mutant alleles, corresponding to more than 50% of the cells carrying a* NRAS* heterozygous mutation. Because tumor samples reflect the average degree of all cell types present in the sample, including normal stromal cells, endothelial cells, and inflammatory cells, the finding that the* NRAS* mutation occurred in more than 50% of cells within the tumor suggests strongly that it is a clonal mutation in these tumors. In the remaining 20% of the FNs, the frequency of mutant alleles was 14–24%. One study reported that these tumors could be classified as clonal neoplasms despite the different histological appearance and that many RAS-positive clonal tumors have been misclassified as histologically nonclonal tumors [17].

In our study, there was no difference in the malignancy rate between FNs diagnosed by CNB and FNAB. The overall malignancy rate of FN and ratio of conventional FN to HCN were consistent with those of our previous study [25].

Our study showed that FNs with* NRAS* mutations had no significant differences in clinicopathological features except tumor size compared with* NRAS*-negative tumors. The presence of* NRAS* mutations was inversely correlated with tumor size, regardless of the presence of malignancy. This finding differs from those of previous studies [15, 26]. This discrepancy might have been caused by the small number of cases and different histological types of tumors in individual series. Further studies across a larger number of cases are needed.

Limitations of the present study include the relatively small sample size and short follow-up period. Surgical specimens were used to test* NRAS* mutation rather than preoperative FNA or CNB specimens. However, most tumors showed a high mutant allele frequency and results of* NRAS* mutation in all CNB specimens were consistent with those in surgical specimens. As* NRAS* mutations occur most frequently in FN and were detected exclusively at codon 61 in exon 3, we only examined the impact of the* NRAS* codon 61 testing on clinicopathological significance. Further studies are needed to clarify the preoperative role of* NRAS* mutations for guiding the surgical approach and their prognostic role in patients with FN.

In conclusion, determining* NRAS* mutation status in FNs is expected to further improve the accuracy of cancer diagnoses and also to help predict cancer risk in thyroid nodules accompanying FNs.

## Figures and Tables

**Table 1:** Clinicopathological features and the *NRAS* mutation status of 123 follicular neoplasms (FNs).

	Patients with a *NRAS* negative FN (*N* = 90)	Patients with a *NRAS* positive FN (*N* = 33)	*P*-value
Average FN size (cm)	2.2 ± 1.5	1.4 ± 0.8	<0.001
Age			
≤45 years	31 (65%)	17 (35%)	0.086
>45 years	59 (79%)	16 (21%)	
Gender			
Female	71 (74%)	25 (26%)	0.710
Male	19 (70%)	8 (30%)	
Final results of FNs			
Benign	52 (83%)	11 (17%)	0.016
Malignancy	38 (63%)	22 (37%)	
Final results of coexisting nodules∗			
Benign	26 (83%)	10 (28%)	0.080
Malignancy	12 (63%)	12 (50%)	
Total risk of malignancy	53% (47/90)	79% (26/33)	0.008

*Of all 123 patients, 60 had one or more incidental nodules in addition to index nodule with a preoperative diagnosis of FN.

**Table 2:** Clinicopathologic features and the *NRAS* mutation status of 60 thyroid cancers with a preoperative diagnosis of follicular neoplasm.

	Patients with a *NRAS* negative thyroid cancer (*N* = 38)	Patients with a *NRAS* positive thyroid cancer (*N* = 22)	*P*-value
Average tumor size (cm)	2.2 ± 1.4	1.4 ± 0.9	0.012
Age			
≤45 years	17 (59%)	12 (41%)	0.464
>45 years	21 (68%)	10 (32%)	
Gender			
Female	26 (62%)	16 (38%)	0.726
Male	12 (67%)	6 (33%)	
Histopathology			
Encapsulated FVPTC	19 (51%)	18 (49%)	
Infiltrative FVPTC	4 (67%)	2 (33%)	
Classic PTC	3 (100%)	0	
FTC	11 (85%)	2 (15%)	
Poorly differentiated	1 (100%)	0	
Capsular invasion			
Negative	13 (50%)	13 (50%)	0.061
Positive	25 (74%)	9 (26%)	
Vascular invasion			
Negative	31 (61%)	20 (39%)	0.329
Positive	7 (78%)	2 (22%)	
Lymph node metastasis			
Negative	25 (66%)	13 (34%)	0.400
Positive	4 (50%)	4 (50%)	

FVPTC, follicular variant of papillary thyroid carcinoma; PTC, papillary thyroid carcinoma; FTC, follicular thyroid carcinoma.

**Table 3:** Clinicopathologic features and the *NRAS* mutation status of 63 benign lesions with a preoperative diagnosis of follicular neoplasm.

	Patients with a *NRAS* negative benign nodule (*N* = 52)	Patients with a *NRAS* positive benign nodule (*N* = 11)	*P*-value
Average tumor size (cm)	2.2 ± 1.5	1.4 ± 0.7	0.010
Age			
≤45 years	14 (74%)	5 (26%)	0.224
>45 years	38 (86%)	6 (14%)	
Gender			
Female	45 (83%)	9 (17%)	0.684
Male	7 (78%)	2 (22%)	
Histopathology			
Follicular adenoma	41 (80%)	10 (20%)	0.355
Nodular hyperplasia	11 (92%)	1 (8%)	

## References

[1] Bongiovanni M, Spitale A, Faquin WC, Mazzucchelli L, Baloch ZW (2012). The Bethesda system for reporting thyroid cytopathology: a meta-analysis. *Acta Cytologica*.

[2] Baloch ZW, LiVolsi VA, Asa SL (2008). Diagnostic terminology and morphologic criteria for cytologic diagnosis of thyroid lesions: a synopsis of the national cancer institute thyroid fine-needle aspiration state of the science conference. *Diagnostic Cytopathology*.

[3] Yoo C, Choi HJ, Im S (2013). Fine needle aspiration cytology of thyroid follicular neoplasm: cytohistologic correlation and accuracy. *Korean Journal of Pathology*.

[4] Nikiforov YE, Ohori NP, Hodak SP (2011). Impact of mutational testing on the diagnosis and management of patients with cytologically indeterminate thyroid nodules: a prospective analysis of 1056 FNA samples. *The Journal of Clinical Endocrinology & Metabolism*.

[5] Kim TY, Kim WB, Song JY (2005). The *BRAF*
^V600E^ mutation is not associated with poor prognostic factors in Korean patients with conventional papillary thyroid microcarcinoma. *Clinical Endocrinology*.

[6] Fukushima T, Suzuki S, Mashiko M (2003). BRAF mutations in papillary carcinomas of the thyroid. *Oncogene*.

[7] Cho U, Oh WJ, Bae JS (2014). Clinicopathological features of rare BRAF mutations in Korean thyroid cancer patients. * Journal of Korean Medical Science*.

[8] Nikiforova MN, Lynch RA, Biddinger PW (2003). RAS point mutations and PAX8-PPAR*γ* rearrangement in thyroid tumors: evidence for distinct molecular pathways in thyroid follicular carcinoma. *The Journal of Clinical Endocrinology and Metabolism*.

[9] Nikiforov YE (2008). Thyroid carcinoma: Molecular pathways and therapeutic targets. *Modern Pathology*.

[10] Volante M, Rapa I, Gandhi M (2009). RAS mutations are the predominant molecular alteration in poorly differentiated thyroid carcinomas and bear prognostic impact. *The Journal of Clinical Endocrinology and Metabolism*.

[11] Garcia-Rostan G, Zhao H, Camp RL (2003). ras Mutations are associated with aggressive tumor phenotypes and poor prognosis in thyroid cancer. *Journal of Clinical Oncology*.

[12] Bos JL (1989). ras Oncogenes in human cancer: a review. *Cancer Research*.

[13] Lee SR, Jung CK, Kim TE (2013). Molecular genotyping of follicular variant of papillary thyroid carcinoma correlates with diagnostic category of fine-needle aspiration cytology: values of RAS mutation testing. *Thyroid*.

[14] Liu R-T, Hou C-Y, You H-L (2004). Selective occurrence of ras mutations in benign and malignant thyroid follicular neoplasms in Taiwan. *Thyroid*.

[15] Vasko V, Ferrand M, di Cristofaro J, Carayon P, Henry JF, De Micco C (2003). Specific pattern of RAS oncogene mutations in follicular thyroid tumors. *Journal of Clinical Endocrinology and Metabolism*.

[16] Fukahori M, Yoshida A, Hayashi H (2012). The associations between ras mutations and clinical characteristics in follicular thyroid tumors: new insights from a single center and a large patient cohort. *Thyroid*.

[17] Gupta N, Dasyam AK, Carty SE (2013). RAS mutations in thyroid FNA specimens are highly predictive of predominantly low-risk follicular-pattern cancers. *The Journal of Clinical Endocrinology and Metabolism*.

[18] An JH, Song KH, Kim SK (2014). RAS mutations in indeterminate thyroid nodules are predictive of the follicular variant of papillary thyroid carcinoma. *Clinical Endocrinology*.

[19] Widder S, Guggisberg K, Khalil M, Pasieka JL (2008). A pathologic re-review of follicular thyroid neoplasms: the impact of changing the threshold for the diagnosis of the follicular variant of papillary thyroid carcinoma. *Surgery*.

[20] Armstrong MJ, Yang H, Yip L (2014). PAX8/PPARgamma rearrangement in thyroid nodules predicts follicular-pattern carcinomas, in particular the encapsulated follicular variant of papillary carcinoma. *Thyroid*.

[21] Jang EK, Song DE, Sim SY (2014). NRAS codon 61 mutation is associated with distant metastasis in patients with follicular thyroid carcinoma. *Thyroid*.

[22] Esapa CT, Johnson SJ, Kendall-Taylor P, Lennard TWJ, Harris PE (1999). Prevalence of Ras mutations in thyroid neoplasia. *Clinical Endocrinology*.

[23] Manenti G, Pilotti S, Re FC, Porta GD, Pierotti MA (1994). Selective activation of *ras* oncogenes in follicular and undifferentiated thyroid carcinomas. *European Journal of Cancer*.

[24] Proietti A, Sartori C, Borrelli N (2013). Follicular-derived neoplasms: morphometric and genetic differences. *Journal of Endocrinological Investigation*.

[25] Min HS, Kim JH, Ryoo I, Jung SL, Jung CK (2014). The role of core needle biopsy in the preoperative diagnosis of follicular neoplasm of the thyroid. *APMIS: Acta Pathologica, Microbiologica, et Immunologica Scandinavica*.

[26] Park JY, Kim WY, Hwang TS (2013). BRAF and RAS mutations in follicular variants of papillary thyroid carcinoma. *Endocrine Pathology*.

